# Interfaces between oncology and psychiatry

**DOI:** 10.1590/1806-9282.2024S129

**Published:** 2024-06-07

**Authors:** Ana Luiza Silva Teles, Leonardo Baldaçara, Antônio Geraldo da Silva, Verônica da Silveira Leite, Ana Lucia Paya Benito, Flávio Veloso Ribeiro

**Affiliations:** 1 Centro Universitário de Brasília – Brasília (DF), Brazil.; 2 Universidade Federal do Tocantins – Palmas (TO), Brazil.; 3 Brazilian Psychiatric Association – Rio de Janeiro (RJ), Brazil.; 4 Universidade Federal de Minas Gerais – Belo Horizonte (MG), Brazil.; 5 Brazilian Society of Psycho-oncology – São Paulo (SP), Brazil.

## INTRODUCTION

Cancer caused nearly 10 million deaths worldwide in 2020, or one-sixth of all deaths. Cancer is a difficult disease with many physical and psychological effects. Pain, weariness, and appearance changes may occur. They may struggle with depression, anxiety, and hopelessness. The sickness and its treatment raise life situations and existential and spiritual questions. The patient and their family and friends suffer during this long and difficult journey^1,2^.

Mental problems are 30–60% more common in cancer patients. In addition, 29–43% of these individuals fulfill mental illness diagnostic criteria^3–5^ with 1.28 more chance than controls^6^. Depression, adjustment difficulties, anxiety disorders, and delirium are common in these people. Advanced disease patients have a greater frequency and worse prognosis for these disorders^3–5^. Unfortunately, the occurrence of these common disorders, which have the potential for successful treatment, is underestimated and undertreated in cancer patients. Only 10% of these persons are referred for mental health services, according to empirical evidence^3–5^. The problems of stigma and discrimination, poorer dignity, poorer health behavior, and lack of integration in health-care services for people with severe mental disorders need to be addressed and solved in cancer care^7–9^.

Interdisciplinary collaboration across medical disciplines is needed to advance cancer research and improve clinical care due to its complexity. Allowing cancer patients to communicate their fears might induce psychological distress management. Cognitive-behavioral therapy, crisis intervention, problem-solving, supportive, and group psychotherapy have been shown to reduce distress and improve the quality of life in cancer patients^10^. Psychotropic drugs and a psychiatrist are needed for severe and long-lasting symptoms. Mental condition differential diagnosis requires a thorough and specialized examination to distinguish between main and secondary causes^4^.

Despite the importance of recognizing and correctly managing mental disorders in cancer patients, there is still less information in the literature on the subject. Therefore, this article aims to present a narrative review regarding the interfaces between oncology and psychiatry, in addition to discussing how the psychiatrist can assist the oncologist and other professionals who deal with oncological diseases in the correct management of mental disorders with a focus on in improving the prognosis and quality of life.

## METHODS

A narrative review was carried out using the following keywords according to Mesh: oncology AND mental disorders. There was no restriction by language or date. The following articles were included: meta-analysis, systematic and non-systematic review, guidelines, clinical trials, cohorts, case-control, and cross-sectional studies. The following were excluded: case reports, case series, editorials, letters to the editor, and abstracts in event annals. Based on their technical knowledge and experience, the authors selected articles for inclusion in the final text for convenience.

## RESULTS

Many cancer patients have psychological anguish after their diagnosis and treatment, regardless of stage. Distress includes unfavorable experiences impacted by cognitive, behavioral, emotional, social, spiritual, and physical variables. It can impair cancer management, including symptoms and therapy. Distress ranges from vulnerability, sadness, and anxiety to severe suffering and psychological and social impairment, which may indicate a mental disease^11–13^. Stress can result from cancer diagnosis and the many changes that occur during treatment and afterward. Despite advances in cancer detection and treatment, the prevalence of long-term side effects outweighs the efficacy of cancer treatments in improving survival rates across all age groups. Patients' everyday activities are hindered by weariness, discomfort, worry, and sadness^11–13^.

Those with a history of mental illness, depression, or substance abuse are more likely to experience moderate or severe discomfort. Cognitive impairment, major concomitant diseases, uncontrolled symptoms, communication issues, and social barriers increase risk. Younger age, living alone, having young dependents, and earlier trauma and abuse—physical, sexual, emotional, and verbal—are social challenges and risk factors. Understanding cancer genetics is linked to emotional and cognitive distress. Distress has been linked to non-adherence to oncological treatment, increased difficulty making treatment decisions, increased medical appointment frequency, prolonged hospital stays, decreased quality of life, decreased surveillance examination participation, reduced physical activity, and limited smoking cessation progress^11–13^.

Support from a psychiatrist and differential and early diagnosis help with a better prognosis and improved quality of life and can prevent the emergence of a mental disorder or its worsening when it already exists.

### Management of mental disorders in oncology

#### Delirium

Neurocognitive impairment caused by brain dysfunction is sometimes called delirium. Changes in consciousness occur suddenly in this state. Patients may develop the neurocognitive and behavioral syndrome at any stage of cancer development, including at diagnosis. This condition may result from cancer, medicine, surgery, or nonmalignant diseases including myocardial infarction^14,15^. Advanced-stage cancer patients have a 90% chance of developing delirium in their final hours, days, and weeks. The most used screening tool is the Confusion Assessment Method (CAM). Delirium's four main symptoms—sudden start and fluctuating course, lack of attention, decreased cognitive functioning, and consciousness changes—form the basis for CAM diagnosis. The CAM method requires criteria (1), (2), and (3) or (4) to diagnose delirium^14,15^.

Delirium is treated with pharmaceutical and nonpharmacological methods. Doctors, nurses, and caretakers must collaborate on nonpharmacological treatments. Healthcare practitioners try to alleviate patient stress while guaranteeing patient safety and integrity. Patient and staff safety must always come first. To prevent patient, caregiver, and staff harm, lines and catheters must be repaired immediately^15,16^. A recommended routine includes bed exercises and walking. Physical constraints can worsen symptoms and cause psychological distress; thus, they should be minimized. Patients' needs, including toilet access, must be met immediately. Superfluous procedures and annoying inputs such as light, noise, and bustle should be reduced. Eyeglasses and hearing aids can remedy visual or auditory impairments. To ensure comfort and familiarity, a familiar person should be positioned near the patient. Family and carers should be informed about delirium and its progression^15,16^. This effort educates caregivers and family members on patient support and agitation management. Medical experts should deliver this instructional intervention. Before starting medication, delirium's multiple causes must be identified and treated. Opioids and other risky drugs should be avoided. To eliminate kidney metabolites, infections and hydration must be treated. Antipsychotics including olanzapine, quetiapine, and aripiprazole may help cancer patients with delirium by increasing calm^15,16^.

#### Anxiety disorders

Threats cause psychological and bodily anxiety. Cancer is a life-threatening condition that can cause worry in many individuals. In one research, 77% of 913 patients experienced anxiety within 2 years of medication. Anxiety disorders have several symptoms. Quantitatively excessive reactions, such as anxious adjustment disorder, often occur within a month of stress^17,18^. Generalized anxiety disorder (GAD) requires more symptoms than anxious adjustment disorder and symptom persistence for 6 months. In these conditions, anxiety often seems free-floating, without a precipitant or intensification pattern. Panic disorder causes anxiety to build to a peak. Phobic anxiety only responds to certain triggers, causing anticipatory avoidance. Medical facilities and therapies can cause phobias, and animal and social phobias may precede cancer. A descriptive classification of anxiety disorders is common. Regardless of its qualities, aberrant anxiety caused by an organic stimulation is called organic anxiety. Drugs like interferon can cause organic anxiety in cancer patients. Depression and anxiety might arise. Cancer specialists are responsible for diagnosing cancer patients' anxiety. Cancer specialists are still poor at recognizing and treating patients with mental disorders. Many questionnaires have been used to measure psychological discomfort and depression in cancer patients. All these procedures perform poorly when compared with standardized psychiatric interviews, and their use does not improve depression or anxiety outcomes. The explanation for these poor results is inadequate. Several self-report surveys measure anxiety specifically^17,18^. However, their relative effectiveness in detecting elevated anxiety levels is unclear. Identifying high-risk populations may help discover anxiety disorders. Younger people, women, and the disadvantaged are more likely to worry. Anxiety symptoms rise following a cancer diagnosis but decrease with time. Several contextual variables affect cancer patients' anxiety. Cancer research has traditionally examined anxiety as a continuum rather than pathological levels, making it unclear how cancer-related conditions affect anxiety disorders or adaptive normal anxiety. People with such symptoms must consult a doctor. Scales aid identification. The nosological diagnosis should guide treatment, which includes psychotherapy, with cognitive-behavioral psychotherapy being the most common, and psychotropic medicines of various durations^17,18^.

#### Mood disorders

Mood disorders pose a substantial health and economic burden across the globe^19^. Due to their chronic, often recurrent nature and common pathophysiological pathways, mood disorders have been associated with a host of physical conditions and illnesses, including cardiovascular disease, diabetes, gastroesophageal reflux disease, asthma, arthritis, and bone fracture. Moreover, mortality rates among those with mood disorders have been estimated to be 35% greater than in the general population, with most of these deaths due to comorbid chronic physical conditions. In the case-control study (n=807), mood disorder was documented for 18 of the 75 (9.3%) cancer cases and among 288 controls (24.0% vs. 39.3%)^20^.

Suspicion should arise not only in the presence of mood symptoms (e.g., hypothymia, euphoria, or mixed state) but also in a previous history of mood disorder. Reduced pleasure, difficulties with sleep, changes in appetite, reduced expectations about the future, and ideas of death (with or without planning) may suggest the presence of depression. Increased energy, reduced need for sleep, accelerated thinking, and grandiosity may suggest mania. When conducting the case, it is important to share with the psychiatrist the investigation of possible primary or secondary causes. Examples of the latter include medications, the inflammatory process itself, and hormonal changes (such as the euphoria caused by increased serotonin production in carcinoid tumors or the effects of thyroid hormone supply in preventing recurrence in thyroid neoplasms)^21–23^. Treatment will depend on the diagnosis: depressive disorder (psychotherapy and antidepressants), mania, or mixed state (mood stabilizers and/or atypical antipsychotics)^22,24,25^.

#### Psychotic disorders

Studies have noted that people with schizophrenia or mental disorders are most diagnosed in advanced stages of cancer^4,26,27^. Some symptoms of schizophrenia can emerge secondary to brain tumors and chemotherapy and can be confused with symptoms of delirium^4,26,27^. The preexisting or recent-onset psychosis can have a negative impact on the quality of care, continuity of care, and reaching remission as it is noted that a significant number of people are lost to follow-up in 1 year^4,26,27^. The quality of care is further poor in the homeless and institutionalized psychiatric patients. Treatment involves the use of antipsychotics; however, it is important that the team is aware of the limitations of these patients who have a distorted sense of reality^28^. Such patients will need support from their family members to make decisions. Delusional symptoms should not be confronted directly. At the same time, careful guidance is necessary regarding the state of health and the steps of the entire treatment^4,26,27,29^.

#### Suicide behavior

Mental problems such as mood, substance use, psychotic, personality, and anxiety disorders can lead to suicidal behavior. Suicide risk among cancer patients who have mood disorders or anxiety and somatoform disorders is higher than for those without mental disorders^30^. A unified framework for describing suicidal conduct must include thought, planning, and attempt^31–33^. This improves situational management. Risk factors for suicide behavior must be categorized by the individual's condition, genetic predisposition, demographics, psychological variables, physical well-being, and health status, including chronic diseases. Also, the person's history of suicidal conduct, including non-suicidal self-harm, should be evaluated^31–33^. The examination of a mental disorder requires a safety plan that includes counseling, research, and monitoring to protect the individual. Suicide-risk persons must be monitored. Hospitalization may be necessary to protect their health in high-risk scenarios such as repeated attempts or a set strategy^31–33^.

## DISCUSSION

As previously discussed, the prevalence of mental illnesses in cancer patients is significant, underscoring the criticality of effectively managing these conditions. The presence of stress associated with illness is a risk factor that requires attention, as it has been identified as a contributing element in the onset of mental disorders. There is a limited body of literature pertaining to this subject, and the current knowledge on differential diagnosis and therapy draws upon the same information utilized for other patient populations. When formulating a treatment plan, it is crucial to carefully evaluate any secondary reasons (such as drugs and clinical disorders) to see if they may be reversed before taking any psychiatric medication. The management of such circumstances typically entails psychotherapy or pharmacotherapy and necessitates the involvement of a psychiatrist collaborating with the oncology team.

## CONCLUSION

The prevalence of mental illnesses among individuals diagnosed with cancer is significant, necessitating the crucial involvement of a psychiatrist in their treatment. These subjects exhibit considerable research potential, as there is a dearth of specialized investigations within this particular cohort.
